# Medical Opinion and Movement

**Published:** 1909-06-26

**Authors:** 


### Medical Opinion and Movement.

THE Administration of Mercury in severe cases of Syphilis by intravenous injections may be said to be still *sub judice*. The chief objection to this method is the localised phlebitis that it is liable to induce. Dr. G. P. Crume reports, in the *Journal of the American Medical Association*, that he has carried out more than 200 injections without any accident or complication. He uses a one per cent. solution of cyanide of mercury and injects one to three cubic centimetres every day or every other day, according to the tolerance of the patient and the severity of the disease. He considers intravenous injections are much more rapid in their action than intramuscular, and advocates this mode of administration especially in cases of cerebral, ocular, and visceral forms of the disease. He uses the glass syringe of Luer, with a chromic platinum needle, and he selects one of the veins on the dorsum of the hand for injection. The arm is bandaged tightly above the wrist to arrest the return circulation, and the needle mounted on the syringe is introduced into the most prominent vessel in a parallel direction. If the needle is well within the vessel, blood appears in the syringe. The bandage is then removed and the injection made.

---

THE Lingual Tonsil, and the troubles that may arise from its Hypertrophy, appear to have received inadequate attention at the hands of medical practitioners; at any rate, such is the opinion of Dr. Maurice, who contributes an article on the subject to the *Journal de Médicine*. The lingual tonsil is the fourth tonsil of the ring of Waldeyer, of which the third gives rise to the adenoid vegetations of the nasopharynx. It is situated in the vertical portion of the tongue, between the V of the calciform papillæ and the glosso-epiglottidean fossæ. It is well developed in children and tends to disappear at puberty. The atrophy commences usually at the middle, and consequently gives it a bilobed appearance. According to Dr. Maurice, fifty per cent. of the patients who consult him for laryngeal or pharyngeal troubles present a hypertrophy of this tonsil, and a large number of them only require treatment for this affection. The hypertrophy may not in itself give rise to symptoms unless congestion and inflammation induced by cold supervene. The so-called " irritable " or " nervous " cough is often caused by a morbid condition of this lingual tonsil, and treatment on this line effects a speedy cure. The tonsil can be examined and its condition ascertained by the finger, but laryngoscopic examination is, of course, much more satisfactory. It frequently presses upon the epiglottis, so that the latter cannot be sufficiently raised to get a view of the larynx. The condition is best treated by the application of the electric cautery. If the hypertrophy is very considerable, the gland may be removed by a Lennox-Brown tonsillotome.

---

EXTENSION or " Stretching " of the Sciatic Nerve is a well-recognised surgical method of treatment for sciatica, and its efficacy has been explained on the supposition that it frees the nerve from adhesions formed around it as a result of a perineuritis. Dr. Paul Carnot, writing in *Le Progrès Médical*, recommends a method of continued extension similar to that adopted in cases of fractured femur. A stirrup of diachylon plaster extending beyond the foot is fixed to the leg by encircling bands of plaster, and a small piece of wood is fitted in the stirrup, from which a cord passes over a pulley at the foot of the bed, and to this is attached a weight. By raising the foot of the bed counter-extension is obtained by the weight of the body. In the case reported by Dr. Carnot, two kilos did not produce sufficient extension to relieve the pain, but three kilos immediately effected this. In this case, every other means of treatment had been adopted previously without result, before extension was tried. Relief from pain only lasted at first so long as the extension was applied; but after continual extension for some days the weight could be removed for several hours without recurrence of the pain. In course of time the extension was only applied at night, and the patient was able to get up in the day. At the end of fifteen days the extension was dispensed with altogether, and the patient only complained of a sort of dull ache on fatigue. Apart from the cure that the treatment appears to have effected in this particular case, the method is simpler, more rational, and much less heroic than the usual methods of " nerve stretching," and has therefore much to recommend it.

---

AN interesting account is given in the *Zentral Blatt für Chirurgie* of the third attempt to carry out Trendelenburg's Operation for Embolism of the Pulmonary Artery. The first two operations were performed by Trendelenburg and his assistant Sievers at Liepzig. This third attempt was made by Dr. Krüger, of Iena. The patient was a woman, aged thirty-three, who had undergone operation for radical cure of a right inguinal hernia. All went well till suddenly, ten days after the operation, symptoms developed indicating an extensive pulmonary embolism; rapid pulse, dyspnœa and cyanosis. Preparations for operation were rapidly made, and with a few whiffs of chloroform, a flap was raised over the second to the fourth costal cartilages, the cartilages divided just within the costochondral articulations, and the whole laid back on the sternum. The edge of the left lung was seized with forceps and fixed to the outer edge of the incision, and the pericardium opened. The pulmonary artery appeared hard and obstructed. An indiarubber tube was passed behind the artery in the transverse sinus of the pericardium and two pairs of Kocher's forceps applied to the artery. Between these the artery was incised for two centimetres and a large clot was extracted. The heart ceased to beat and the pupils became dilated. The incision was provisionally closed with forceps and the heart massaged. The heart quickly recovered. A further clot was then removed, and behind it black blood flowed in abundance. The heart again ceased to beat. The same proceeding was repeated and the heart beat again. The artery was sutured and the operation completed. Afterwards the pulse was good and cyanosis disappeared. The condition remained favourable for two days, but lung trouble supervened and the patient succumbed on the fifth day. Autopsy showed suppurative pleurisy of the left side, involving also the mediastinum and thoracic wound. Dr. Krüger attributes this to the haste with which the preparations for operation were necessarily made, and it seems possible that the operation might otherwise have proved successful.

---

Among the causes of Pericolitis, chronic constipation certainly plays a great part. Chronic enterocolitis, hæmatogenous infections, nervous and so-called functional intestinal disturbances must also be recognised as ætiological factors. With regard to the question of chronic constipation the author touches upon the interesting and formerly much debated subject of stercoral typhlitis, *i.e.* a perityphlitis unaccompanied by any lesion in the appendix. With regard to the treatment the author lays stress on the judicious employment of aperients, especially in early cases. In old and neglected cases, purgatives must be very cautiously used, and the question of ultimate surgical interference must be considered. Local applications of cold or hot compresses over the area of tenderness will ease the patient. Rest in bed, and a light—not necessarily wholly fluid—diet are indicated, and in every case the differential diagnosis must be carefully gone into before it is accepted that the case is one of pericolitis. There can be little doubt that at present many cases of this condition are erroneously diagnosed and treated as cases of appendicitis.

---

Pancreatic Cysts are not of everyday occurrence, and this condition in young infants must be extremely rare. Dr. Maxwell Telling and Mr. J. F. Dobson report such a case in a child which came under observation at the age of eleven months for abdominal distension: this symptom the parents had noticed for the preceding two months. The infant had no symptoms of illness, and the presence of a palpable tumour above the umbilicus was so obscure that it was not appreciated by many of those who saw the case. During the next month the baby gradually lost ground and weight, whilst the circumference of the abdomen increased. It was accordingly operated upon, and under chloroform an incision was made mesially above the umbilicus, when a large smooth-walled cyst appeared between the stomach and colon. This turned out to be of pancreatic origin: it was opened and drained. The contained fluid amounted approximately to a pint and a half; it was alkaline, of sp. gr. 1025, contained no sugar, but went nearly solid on boiling, owing to coagulation of albumin. No ferment action was obtainable, there was no evidence of hydatid disease, and the cyst wall was lined by columnar epithelium. As for the patient, the sinus healed in five weeks: despite temporary set-backs in convalescence, the child ultimately did very well, and is now in perfect health.

The viability of the Bacillus Typhosus, as excreted under natural conditions by the "chronic carrier," has been carefully investigated by Major J. C. Morgan and Captain D. Harvey, whose conclusions are published in the *Journal of the Royal Army Medical Corps*, and are of much interest in connection with attempts to prevent the spread of the disease by such patients, commented on in our columns last week (The Hospital, June 19, 1909, p. 300). These observers have experimented with the urine and fæces of two soldiers who were passing enormous numbers of bacilli, respectively eight years and six months after their illnesses. It is shown that micturition pollutes the soil for a maximum period of about 24 hours. After that time no typhoid bacilli can be isolated from any portion of the contaminated ground, whether it has been exposed to the sun or is in the interior of a dark hut. On the other hand, when towelling was soaked in urine containing 50,000 bacilli per c.c. the bacilli were recovered from a piece kept in the dark for eleven days afterwards, whereas a piece exposed to light was no longer infective at the end of four days. In water and in milk the life of the bacillus seems to be extremely short, a few hours being sufficient to render sterile these fluids contaminated directly with urine rich in bacilli. Urotropine in 10-grain doses had absolutely no effect on the bacilluria in the patient on whom it was tried. In dry earth methods of disposal of fæces the bacillus typhosus can be readily recovered up to a week, and it can exist in the interior of a dry fæcal mass for as long as eighteen days.

---

A most unusual case of apparent recovery from Diabetes Mellitus in a boy of 10 years of age was shown at a recent session of the New York Academy of Medicine by Dr. Heiman. The patient when first seen had for five months suffered from thirst, loss of weight, frequency of micutrition, and increased appetite. He was passing 5,700 c.c. (about 10 pints) of urine of specific gravity 1032, with 5 per cent. of sugar; acetone and diacetic acid were both present. At first a fair quantity of carbohydrate food was allowed, but as the diacetic acid did not disappear, the diet was reduced to meat, fish, and a few bulky vegetables such as cabbage, which contain but small quantities of starch. The boy to satisfy his hunger ate enormous quantities of these foods. Bicarbonate of soda, atropine, and olive oil were the drugs ordered; the sulphate of atropine was given three times a day, and the dose increased very gradually up to $\frac{1}{60}$ grain at a time. Under this régime the boy gained weight, lost both sugar and diacetic acid, and now passes but 2,000 c.c. of urine daily. At the same time the least indiscretion in the direction of excess of carbohydrate is followed by immediate glycosuria; 29 grammes a day is as much as can safely be allowed. Notwithstanding the results of treatment, it was considered that the ultimate prognosis must be given with great reserve. Still, diabetes at this age is so uniformly serious and progressive that the case must be regarded as a most remarkable one.

THE whole subject of Cæsarean Section for Placenta Prævia is discussed by various writers in the *American Journal of Obstetrics*. In the main the verdict is unfavourable, though not unanimously so. What seems to have weighed with most of the contributors is the maternal mortality of this operation, which is given as 20 per cent. in placenta prævia cases. It is admitted that a somewhat better prognosis for the fœtus attends abdominal section, but most of the obstetricians report maternal mortalities varying from 3 to 6 per cent. by conservative methods, and this difference is quite rightly held to be decisive against Cæsarean section. One writer, however, Dr. Fry, points out that in many of the 20 per cent. of fatal cases there were quite obvious contra-indications of the operation, and he shows by a table that since 1905 the figures show an enormous improvement in this respect. Dührssen's vaginal Cæsarean section is favourably mentioned by more than one writer as of value in certain special cases, but on the whole the consensus of opinion is in favour of slow dilatation of the cervix until one or two fingers can be admitted, followed by Braxton Hicks' version and by hydrostatic bags if necessary. Emphasis is laid on slow delivery unless hæmorrhage has already been very grave; and accouchement forcé, Bossi's dilators, and rapid extractions in general are condemned almost by everyone. This approximation on the part of obstetricians to British rather than Continental teachings is interesting, especially during the present age of operative midwifery.

---

TUFFIER recently read a paper before La Société de Chirurgie upon the principles which should govern the hot-air treatment of Diabetic Gangrene. Several years of clinical investigation have convinced him that the results attending this line of treatment depend upon the temperature of the air used, and vary according to whether this is projected on to the wound under pressure or the limb placed in a warm atmosphere. Air raised to a temperature of between 35 deg. and 40 deg. C. is particularly useful in cases in which gangrene is imminent, but not definitely declared. When, however, sloughing has commenced, the best results are obtained with air heated to between 300 deg. and 500 deg. C., delivered under pressure. The author merely claims that his rules and suggestions will improve the line of treatment first proposed by Guyot in 1840, and extend its range of application and utility.

---

SURMONT reports a case in which Aortic Insufficiency followed a difficult labour, and seemed to be caused by it. The patient, aged 41, after a labour lasting 48 hours, was delivered with forceps. There was no history of infectious fever, gout, rheumatism, or alcoholism, and the cardiac symptoms all dated from this, her second delivery. Ever since this, which occurred two years ago, the patient has been subject to vertigo and palpitations, and now presents the characteristic physical signs of aortic insufficiency. Her cardiac lesion is therefore included in the category of valvular ruptures, due to violent effort. The fact that at the time of the accident she felt no retro-sternal pain, which is a characteristic symptom of this occurrence, scarcely prejudices the accuracy of the diagnosis, since the absence of this phenomenon might well be explained by the co-existence of labour-pains.

---

A RECENT number of the *Therap. Monatshefte* contains an article by Ehrenayer and Stein on the action of Iodides with reference to Arterio-sclerosis. Iodine preparations only act after liberation of their ions, which are an energetic protoplasmic poison. Cells, the resistance of which is in any way diminished, are thus easily destroyed by the iodide. In arterio-sclerosis the combination of the ion with cells of diminished vitality favours the elimination of these latter. The stimulus must always be maintained equal, an effect which can only be secured by progressively increasing the dose. In addition the drug acts in opposition to the effects of alcohol, which increases the viscosity of the blood. When undergoing treatment with iodides, organic acids should be excluded as far as possible from the diet, as they tend to liberate free iodine. For the same reason milk, which readily gives rise to lactic acid, should be avoided, and it is good practice to give alkaline salts with iodides. The constant fear of producing iodism is therefore due to a therapeutic fallacy, and the notion that certain organic compounds of iodine produce no toxic effects is not borne out by experience. Widely advertised compounds, supported by glowing assertions, give no results which cannot be obtained by progressive doses of inorganic iodides. Moreover, the iodine content in some of these compounds is extremely low. The authors are of opinion that alkaline iodides are preferable, and state that their action is increased by administering several of them together—*e.g.* sodium and potassium iodide. Iodism is the inevitable reaction of iodides. Excessive reaction can be controlled with ease, but it should not be entirely suppressed. An efficient therapeutic effect can only be obtained by a prolonged administration of progressively increasing doses.

---

CHAMPEAUX, of Lorient, in *La Revue Hebdomadaire de Laryngologie* publishes a study of the symptomatology, pathology, and treatment of Sneezing. It has long been a popular notion that rubbing the root of the nose and tapping the frontal region will diminish the sensation of irritation before an attack of sneezing, a fact which Katzenstein explains by the existence of a centre for sneezing in the frontal lobe. Himself a neuro-arthritic and subject to spasmodic rhinitis, the author has personally tried frontal tapping, friction, and massage at the onset of an attack of sneezing, and has found that the most successful of all such measures in his own case is a gentle massage over the frontal region with the palmar surface of the fingers of both hands, commencing at the temples and carried towards the middle line, but not in the opposite direction. This manœuvre must be repeated several times in succession until relief is obtained, and is best performed by a second person.

